# From orogeny to rifting: insights from the Norwegian ‘reactivation phase’

**DOI:** 10.1038/s41598-020-71893-z

**Published:** 2020-09-09

**Authors:** Gwenn Peron-Pinvidic, Per Terje Osmundsen

**Affiliations:** 1 NGU Geological Survey of Norway, Leiv Eirikssons vei 39, 7040 Trondheim, Norway; 2 Department of Geoscience and Petroleum, Norwegian University of Science and Technology (NTNU), 7491 Trondheim, Norway

**Keywords:** Solid Earth sciences, Geodynamics, Geology, Tectonics

## Abstract

Based on observations from the Mid-Norwegian extensional system, we describe how, when and where the post-Caledonian continental crust evolved from a context of orogenic disintegration to one of continental rifting. We highlight the importance of a deformation stage that occurred between the collapse mode and the high-angle faulting mode often associated with early rifting of continental crust. This transitional stage, which we interpret to represent the earliest stage of rifting, includes unexpected large magnitudes of crustal thinning facilitated through the reactivation and further development of inherited collapse structures, including detachment faults, shear zones and metamorphic core complexes. The reduction of the already re-equilibrated post-orogenic crust to only ~ 50% of normal thickness over large areas, and considerably less locally, during this stage shows that the common assumption of very moderate extension in the proximal margin domain may not conform to margins that developed on collapsed orogens.

## Introduction

It has been long noticed that most rifts and rifted margins around the world developed on former orogens, and particularly on former suture zones^1^. The pre-rift lithospheric configuration is thus heterogenous in most cases. However, for convenience and lack of information, most conceptual and numerical models envisage the beginning of rifting based on a homogeneously layered lithosphere^2^. In the last decade, numerous studies have focused on the impact of inheritance on the architecture of rifts and rifted margins^3^, and the pre-rift tectonic history has often been revealed as strongly influencing the subsequent rift phases^4^. Within this framework, the definition of the actual onset of rifting, as opposed to previous phases of deformation, is essential. The observation of extensional structures in zones of convergence, the occurrence of abnormally high topography in extending mountain belts and modeling of the behavior of overthickened orogenic crust led to the concept of gravitational orogenic collapse^5–7^. Some workers have assumed that since orogenic collapse is wholly or partly driven by the gravitational potential anomaly^5^, the associated extension will cease once the crust has returned to a normal thickness equilibrium. Others have suggested it can continue indefinitely^8^.

One important question relates to the onset of extension in the overall orogenic cycle^9^. The related deformation is generally related to polarity reversal along orogenic thrusts, ductile to brittle deformation and important crustal thinning with exhumation of deeply buried rocks^10^. The resulting structural template commonly involves metamorphic core complexes, extensional shear zones and detachment faults superposed on inherited thrust assemblages^SPS:refid::bib99^. Thus, in areas of previous post-orogenic extension or collapse, the onset of rifting cannot be simply equated with the onset of extension: extensional geometries can be related to various previous deformation phases. Regarding the formation of rifted margins within that context, the pertinent question will then be at what point in the extensional history does orogenic collapse stop and rifting start?

Conceptually, the proximal domains of rifted margins are often presented with only moderately reduced crustal thicknesses^11,12^. The top basement geometries are typically summarized as series of tilted blocks, bordered by 'Andersonian-type' normal faults rooted in the brittle-ductile transition at mid-crustal levels, accounting for minor amounts of extension (the ‘stretching phase’ of^2^). Thus, orogenic collapse and early rifting are considered to represent very different deformation modes with distinct structural geometries. Within rifted margins, the detachment faults that facilitate large magnitudes of basement thinning and, eventually, deformation coupling and basement exhumation are mainly located in the necking and distal margin domains^13^.

**Figure 1:**
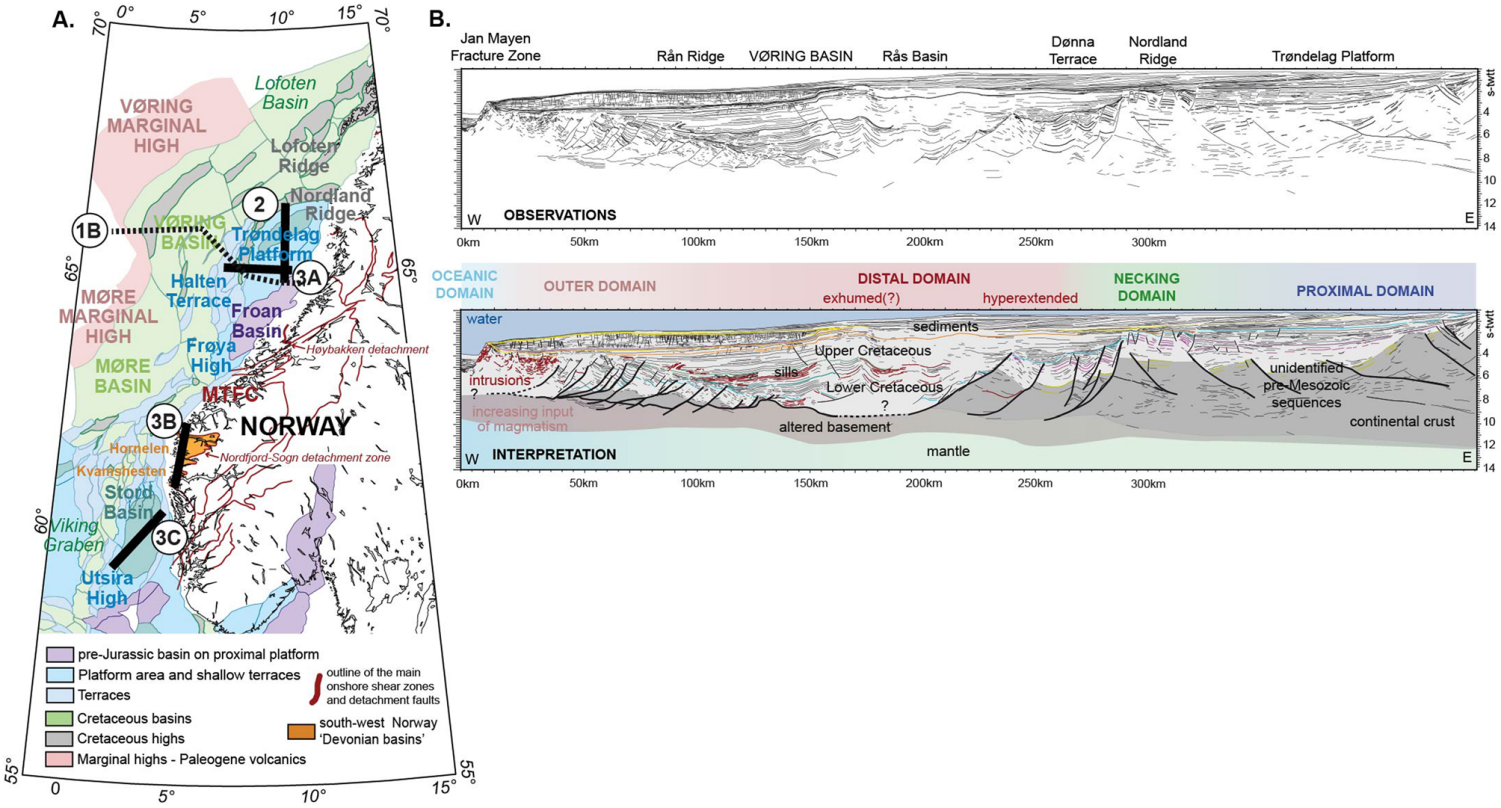
(**A**) Simplified structural map of SW Norway (after^29^). The onshore red lines outline the main mapped shear zones and detachment faults. *MTFC* Møre–Trøndelag Fault Complex. Orange: outline of the 'Devonian basins'. Figure created in Oasis Montaj 9.6 (https://www.geosoft.com/products/oasis-montaj). (**B**) Cross-section illustrating the regional structural context of the Mid-Norwegian rifted margin. Top: line drawing of all observable features. Bottom: interpretation based on our mapping methodology. (location on **A**). The interpretation is based on seismic reflection, seismic refraction and potential field data (modified after^44,45^).

The Mid-Norwegian margin (Fig. 1) is a key laboratory to study these questions and investigate structural distinctions between the extension related to orogenic collapse and that related to rifting *s.s.*

## The Norwegian case

**Figure 2:**
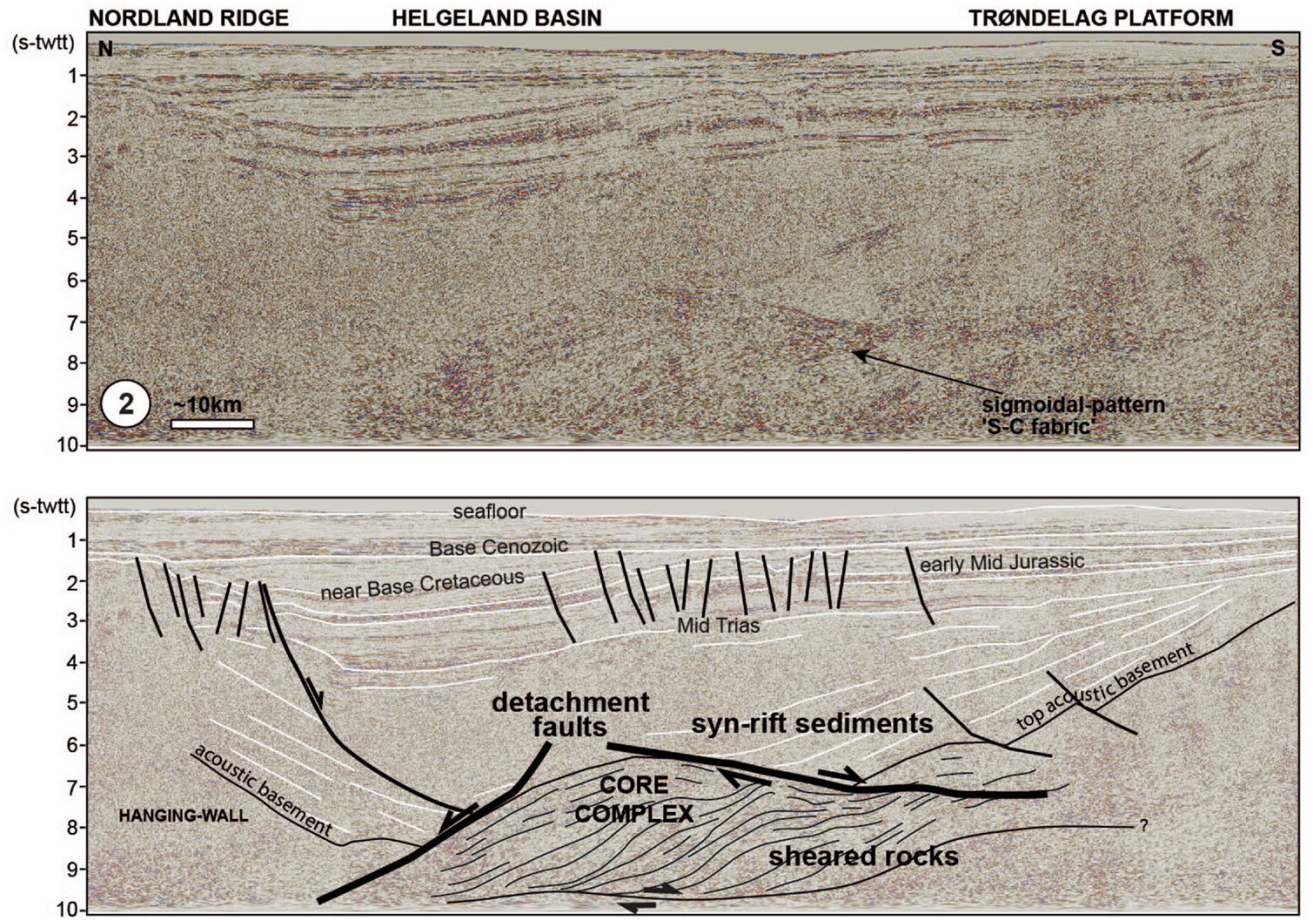
Seismic reflection profile (TGS) from the Trøndelag Platform; in time version (s-twtt), without (top) and with interpretation (bottom) (location on Fig. 1A).

**Figure 3:**
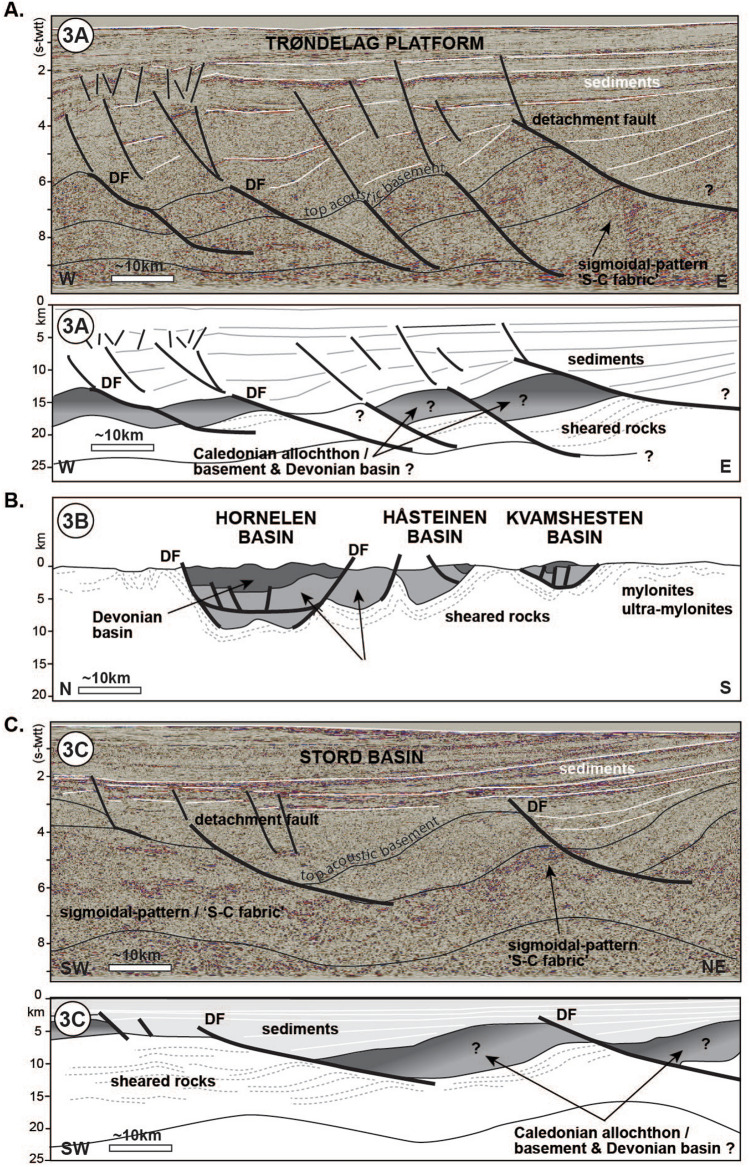
(**A**) seismic reflection profile (TGS) from the Trøndelag Platform; in time version (s-twtt) (top) and with simplified interpreted depth version (bottom) (location on Fig. 1A). Modified from Peron-Pinvidic et al.^24^. (**B**) Schematic cross-section illustrating the structural setting of the south-west Norway 'Devonian basins' (location on Fig. 1A). From Osmundsen and Andersen^15^. (**C**) Seismic reflection profile (TGS) from the North Sea Stord Basin; in time version (s-twtt) (top) and with simplified interpreted depth version (bottom) (location on Fig. 1A).

Onshore outcrops in western and south-western Norway display major extensional shear zones and detachment faults interpreted as related to the collapse of the Scandinavian Caledonides^14^ (Figs. 1, 2, 3). The Nordfjord-Sogn and Høybakken detachment zones juxtapose (ultra-)high pressure rocks in their footwalls with Caledonian nappe remnants and Devonian basins in their hanging walls^15^ (Fig. 3B). The cumulative displacements associated with the detachment zones are interpreted to be on the order of 40–100 km^16^. The detachment zones consist of ductilely sheared rocks that reach thicknesses of about 2–6 km^17^ and capping detachment faults with brittle deformation products^18^. They are therefore major tectonic structures, and have been mapped along the coast of southwest, mid and parts of northern Norway^19,20^. The structures undoubtedly extend offshore, but for an unknown distance^21–23^.

Off the mid- and south-western Norwegian coast, the Trøndelag Platform corresponds to the proximal domain of the Mid Norwegian Vøring margin and the Stord Basin area to the northern parts of the abandoned North Sea rift (Figs. 2, 3). Recent mapping based on modern datasets, has revealed that the crustal thinning of these regions is much more significant than commonly assumed^24^. Both areas are floored at depth by a seismic unit that forms a corrugated surface at a regional scale, with basement culminations that are flanked, and sometimes cut, by extensional detachment faults (Figs. 2, 3A,C). These detachments are segmented, suggesting long-lived multiphase tectonic activity. The unit displays a well-defined fabric with sub-parallel undulating reflectors that show clear deflections and sigmoidal patterns at the level of the basement ridges (Figs. 2, 3A). This sigmoidal-shaped pattern resembles what is often referred to as an 'S-C fabric'—the relative geometry of the *schistosité* vs. *cisaillement* deformation planes in shear zones (e.g.^25^) (Figs. 2, 3A). Similar geometries have been observed on offshore seismic datasets at other rifted margins worldwide (e.g. Uruguay margin^26^) and onshore in various extensional settings outcrops, such as south-west Norway^14^ and Alpine Corsica^27^. These geometries are commonly interpreted as developed in rocks that have been sheared in the ductile deformation field. Based on this, we interpret this proximal basement unit to contain assemblages of mylonitic shear zones, associated with detachment faults. The shear zones show thick and composite structure and are associated with major regional core complexes such as under the Helgeland Basin, the Trøndelag Platform and the Utsira High. The unit above the sheared basement often presents a chaotic seismic facies but can also be characterized by more organized geometries locally, with reflectors produced by stratigraphy and associated onlap relationships, unconformities, prograding geometries and faulting events. Given the underlying rocks interpreted in terms of shear zone and core complexes analogous to the onshore extensional structures, the upper basement geometries may encompass Caledonian basement, pieces of orogenic nappes and Early to middle Devonian basins, similar to those encountered onshore, and/or Late Devonian—Upper Carboniferous sediments (Fig. 3).

## Discussion: when and how does rifting begin?

In the North Atlantic, rifting is usually proposed to begin by Late Carboniferous to Early Permian times^28–31^, involving a series of high angle normal faults generating a graben-type structural environment. As summarized above, mapping permitted by the modern seismic datasets shows that the offshore Norwegian proximal areas—from the Trøndelag Platform down to the North Sea Stord Basin/Utsira High—are floored by a unit of anastomosing shear zones and metamorphic core complexes, associated with long-lived detachment faults^23,24^. Structurally, these geometries appear extremely different from the geometries normally attributed to the early rifting deformation mode in a number of recent models^2^. Important questions thus arise regarding the spatial and temporal boundaries between the two sets of tectonic structures.

### Available time constraints

Published ages, based mainly on Ar–Ar geochronology, help to further constrain the evolution of the region at that particular period. Most come from onshore studies. Fossen^9^ constrained the onset of the orogenic collapse to about 405 Ma, corresponding to the reversal of tectonic transport direction on the basal Caledonian decollement in southern Norway. Eide et al. ^32^ showed that the onshore Høybakken detachment zone was mostly active between 400 and 365 Ma, with the brittle detachment fault cutting through pre-existing extensional mylonites^33^, and Eide et al. ^34^ constrained the crustal exhumation rate to slow down by 380–360 Ma. Gilotti and McClelland^35^ proposed that the Early Carboniferous 345 Ma marks the structural change to rifting *s.s.* for the North-East Greenland conjugate, with high-angle normal faults cutting through basement and Devonian cover. Rotevatn et al. ^36^ recently argued, based on structural data and K–Ar dating work, that activity on inherited post-Caledonian detachment faults and steeper, early rifting faults in East Greenland overlapped in time and that weak, inherited detachments must have played an important role in the earliest phase of rifting.

All these ages constrain the change from collapse to rifting mode to occur in a 30–40 Myrs time interval from the Late Devonian to the Early Carboniferous times. Then, in southwest Norway, reactivation of various major onshore faults has been dated to occur subsequently; like the Nordfjord-Sogn Detachment Fault^37,38^, the Hardangerfjord shear zone^39^ and a series of faults bounding the Bergen arc area^40^ that were reactivated in Late Devonian–Early Carboniferous, Permian and Jurassic-Cretaceous times.

### A new phase of deformation: the 'reactivation phase'

**Figure 4:**
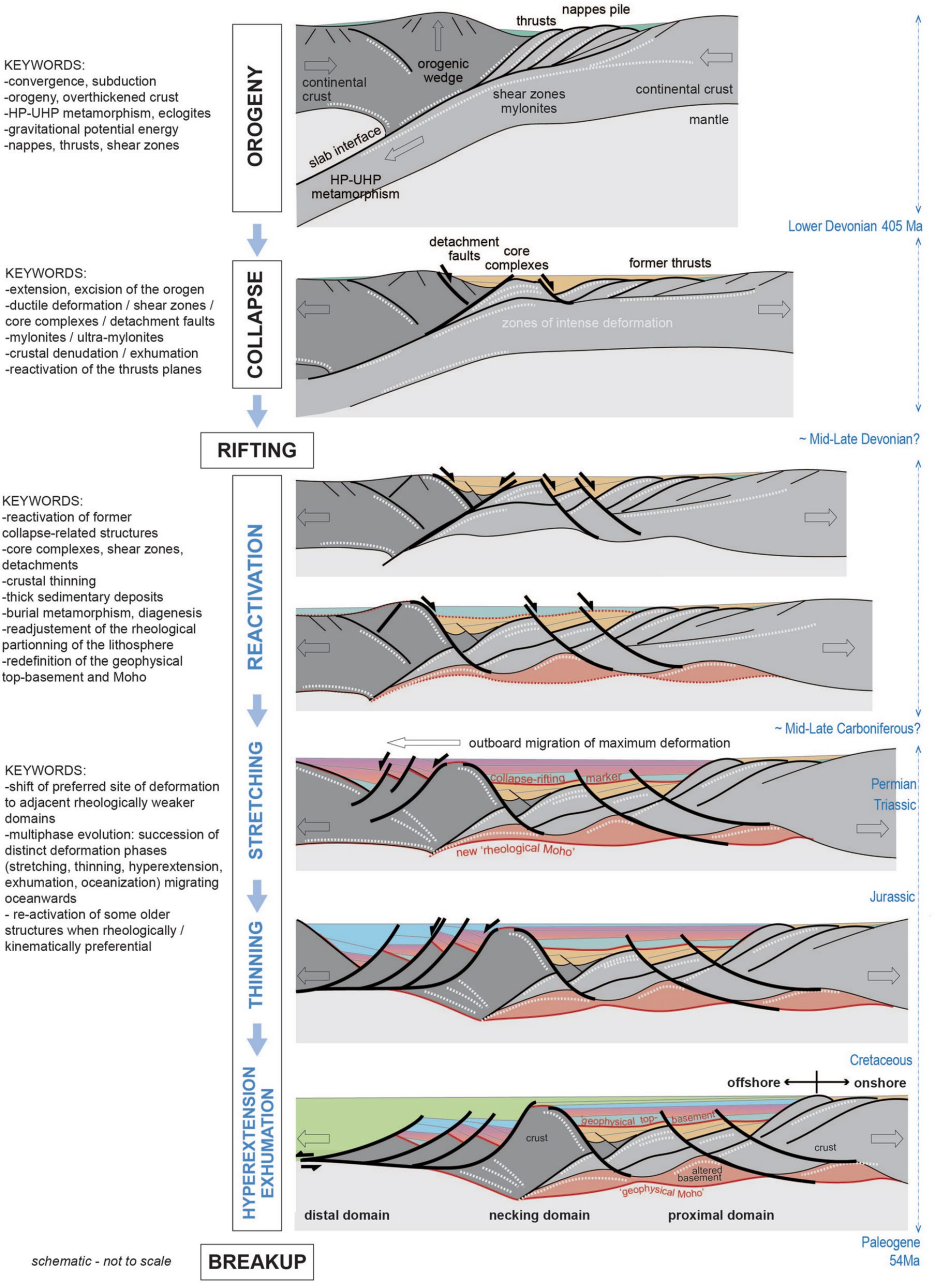
Schematic representation of the structural crustal evolution from an orogenic context to a rift. Left: keywords are listed for each step aiming at summarizing the main structural contexts and geological processes. Right: series of cartoons presented in a rough time evolution from top (orogen) to bottom (rifting). Numbers and geometries are for the Norwegian case. Not to scale. Modified from Peron-Pinvidic et al.^24^.

Based on our observations from seismic reflection data, and on published evidence for reactivation of detachments in East Greenland and Norway, we propose that the transition between extensional collapse and early rifting corresponds to a specific, previously unspecified phase of deformation. This ‘reactivation phase’ (Fig. 4) is interpreted as an important stage in the evolution of rifted margins that initiate on previous orogens. It is a phase of massive re-activation of some of the collapse-related structures with major detachment faulting proceeding to additional crustal thinning (e.g. the Trøndelag Platform). This particular tectonic stage precedes the standard rifting evolution, generating previously unidentified structural geometries in the margin’s proximal domain. In the Norwegian case, the impact for crustal thinning varied along the margin, with the most important reactivation phase extension identified inboard of the Vøring margin.

The transition from the detachment zones to the stretching high angle normal faults is interpreted to have occurred after a period of intense sedimentary influx into the basins associated with drastic crustal thinning. The accumulated sedimentary thicknesses, together with a probable quieter tectonic context, led to a thermal and rheological readjustment of the lithosphere.

Thermal re-equilibration typically includes diagenesis and other petrophysical processes which impact the physical properties of the rocks, at the base of the existing sedimentary basins and at the base of the crust. Onshore south-western Norway, various studies have reported the imprint of a pervasive low grade metamorphism in sub-greenschist facies, which is interpreted to reflect the burial of the Devonian sediments to significant depths^41^, although shear heating and the transfer of remnant heat from the exhumed footwall may also have played a role in these processes^42^. Offshore, similar metamorphism would explain the difficulty in identifying the top of basement in potential field or refraction datasets^43^: because of densification processes related to large basin thicknesses, the upper basement rocks may become indistinguishable from the densified sedimentary layers above.

**Figure 5:**
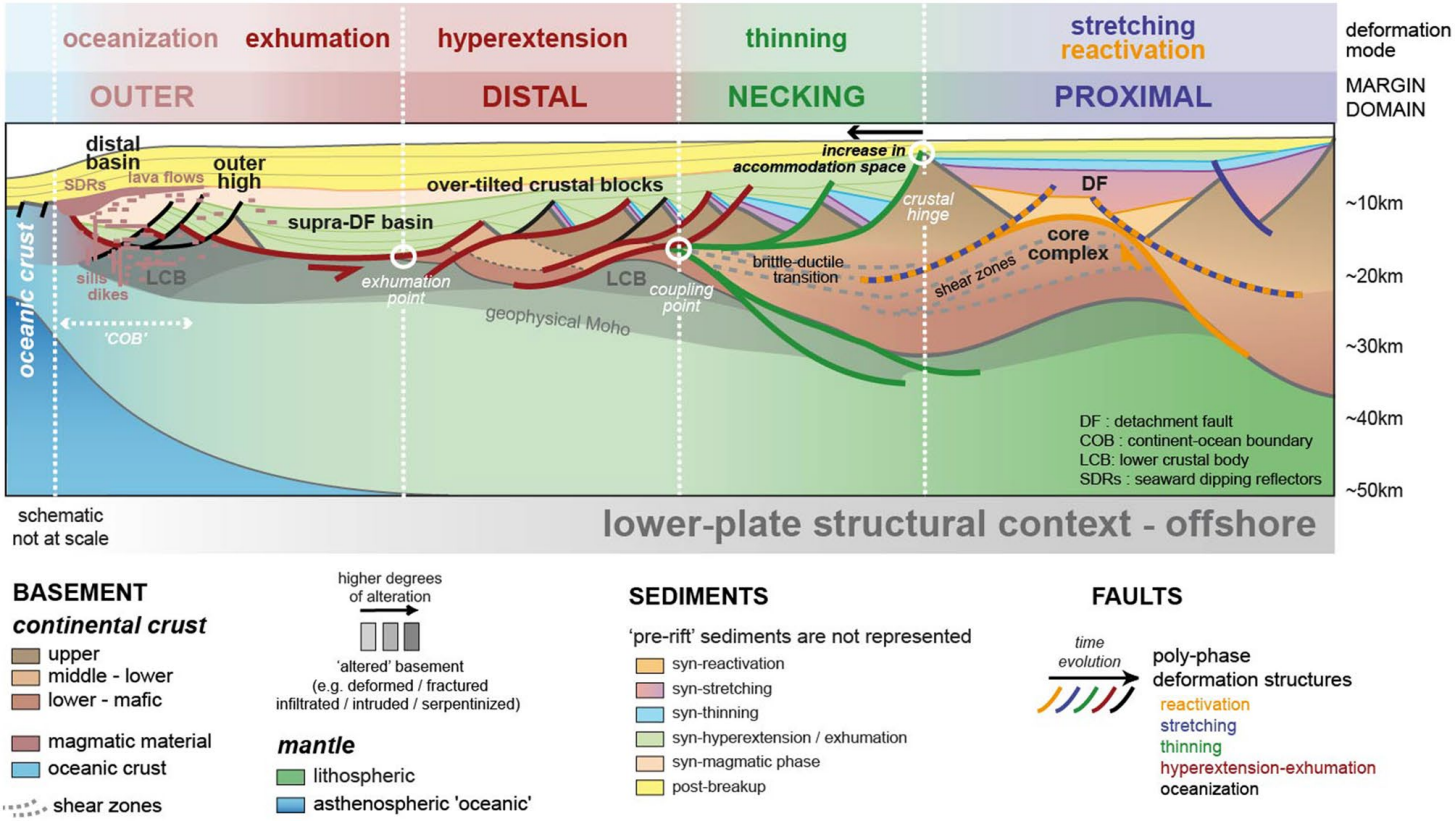
Cartoon summarizing the structural and stratigraphic characteristics of a standard rifted margin developing on an orogen (lower-plate settings). Redrawn after Peron-Pinvidic et al.^12^.

Based on this, offshore mid-Norway, the reactivation phase context is interpreted to have led to a local redefinition of the geophysical basement envelopes (Fig. 4). Within that framework, it is assumed that the lithosphere reached a new equilibrium at that time, that may have induced new decoupled brittle and ductile lithospheric levels, which promoted the development of a different deformation mode during the next tectonic phase: subsequent rifting deformation focused in adjacent less thinned areas, thermally and rheologically more prone to deform. Finally, the extensional system evolved in a succession of distinct deformation phases, migrating oceanwards, as often assumed in conceptual models^2,12^ (Fig. 5).

## Conclusions

Standard rifting models often assume homogeneous lithosphere and 'Andersonian-type' normal faulting for the first phases of rift-related deformation. This may not be representative of rifted margins developed on collapsed orogens: recent observations of the offshore Mid Norwegian and northern North Sea basement geometries attest to a profoundly different structural context with a previously underreported deformation phase. The entire near-coastal onshore and proximal offshore Norwegian region is shown to be floored by a unit of intensively sheared basement, mylonitic shear zones, core complexes and detachment faults that attests to significant crustal thinning. It is proposed that the transitional period between orogenic collapse and rifting corresponds to a specific tectonic phase—the reactivation phase—which includes: (1) re-use of former collapse-related structures, (2) drastic crustal thinning, (3) deposition of thick sedimentary successions and (4) a readjustment of the rheological partitioning of the lithosphere leading to a redefinition of the rheological top-basement and Moho envelopes.
